# Synthesis and redox behavior of Si–Si dimeric 9-methylsilafluorene†

**DOI:** 10.1039/d5dt00097a

**Published:** 2025-03-14

**Authors:** Kelsie E. Wentz, Andrew Molino, George Q. Jiang, Maxime A. Siegler, David J. D. Wilson, V. Sara Thoi, Rebekka S. Klausen

**Affiliations:** a Department of Chemistry, Johns Hopkins University Baltimore MD USA kwentz2@jhu.edu klausen@jhu.edu; b Department of Chemistry, Massachusetts Institute of Technology Cambridge MA USA; c Department of Biochemistry and Chemistry, La Trobe Institute for Molecular Science, La Trobe University Melbourne Victoria Australia; d Department of Materials Science and Engineering, Johns Hopkins University Baltimore MD USA

## Abstract

Lithium-halogen exchange on 2,2′-dibromo-1,1′-biphenyl followed by salt metathesis with 1,1,2,2-tetrachloro-1,2-dimethyldisilane produced the Si–Si linked dimeric 9-methylsilafluorene (1) in 62% yield. Compound 1 was characterized in the solid state *via* single crystal X-ray diffraction, which revealed a highly symmetric structure. The redox behavior of 1 was studied experimentally *via* cyclic voltammetry, where irreversible oxidation and reduction features were observed. Density functional theory was used to further study the electronic structure of 1 as well as its reduced (1˙^−^) and oxidized (1˙^+^) forms. A decrease in bond dissociation energies from the neutral species (+75 kcal mol^−1^) to the reduced species (+43 kcal mol^−1^) supports Si–Si bond homolysis upon reduction, which is consistent with our observed electrochemical irreversibility.

## Abstract 2

The direct synthesis of Si–Si dimeric 9-dimethylsilafluorene, structural characterization *via* single crystal X-ray diffraction studies, and electrochemical redox chemistry with DFT electronic structural analysis is reported herein.

## Graphical abstract

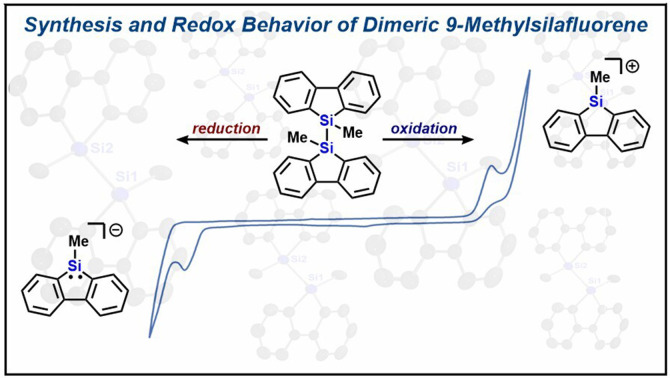

## Introduction

Fluorene is a tricyclic scaffold with a central 5-membered ring fused between two aromatic 6-membered rings.^1^ Fluorene's electronic structure gives rise to luminescence and facile deprotonation or chemical reduction to form an aromatic 14-electron anion^2,3^ which has garnered significant interest in molecular optoelectronics.^4^ Replacement of the central carbon atom with Si gives silafluorene and results in different electronic structure due to variations in how C and Si atoms participate in multiple bonding and aromaticity.^5–8^ The photophysical properties of silafluorene have been extensively investigated for optical and optoelectronic properties.^9–19^

Many studies have focused on linking the silafluorene through the carbon atoms of the π-conjugated backbone and the neutral silafluorene has been widely applied in conjugated materials.^20–22^ Yet linkages through the central Si atom,^23^ such as the dehydropolymerization of 9,9-dihydrosilafluorene reported by Corey *et al.*, and the understanding of electronic properties, redox cycling, and the impact on interactions between the σ and π components of this scaffold are less explored.^23^ This is despite the unique electronic properties of Si–Si chains, which, unlike C–C single bond chains, are both optically and electronically active.^23–27^

**Fig. 1:**
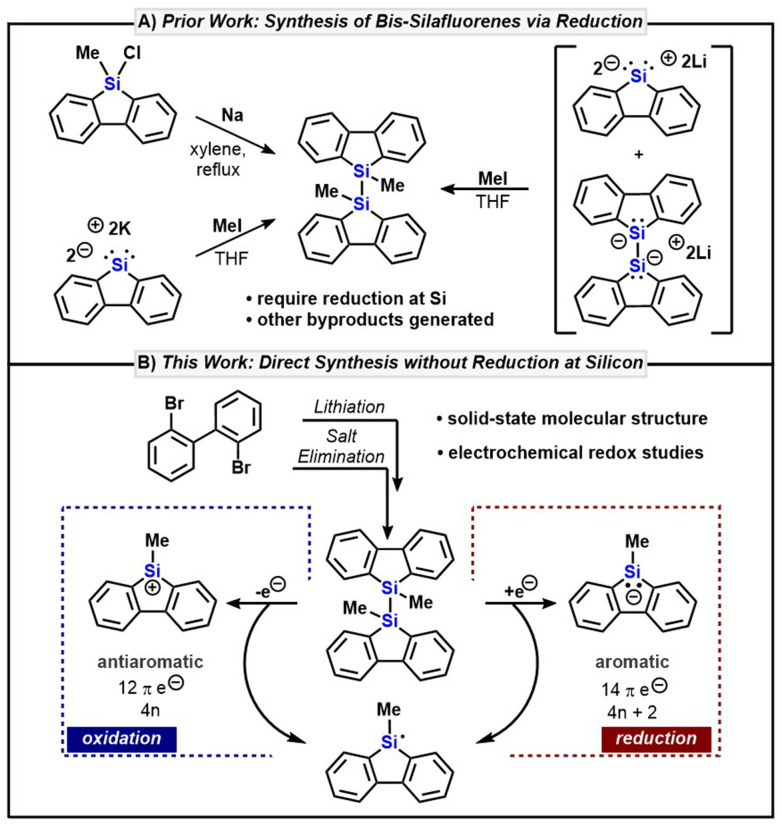
(A) Previous routes to dimeric 9-methylsilafluorene. (B) Direct route to dimeric 9-methylsilafluorene by lithium halogen exchange followed by salt elimination (this work).

Towards a fundamental understanding of the electrochemical properties and reactivity of poly(silafluorene) derivatives, we sought a direct synthesis of the simplest Si–Si linked silafluorene, compound 1. This structure had previously been reported as a product of chemical reduction of 9-chloro-9-methylsilafluorene or methylation of the silafluorene dianion (Fig. 1A).^28–30^ West and Boudjouk separately showed that chemical reduction of dichlorosilafluorene yielded the silafluorenyl dianion. The electronic structure of the dianion appeared to vary with the nature of the chemical reductant. Boudjouk (using excess lithium as the reductant) reported a ^29^Si NMR chemical shift of *δ* −1.09 for the dianion, which was consistent with localization of electron density on silicon.^29^ West (using excess potassium as the reductant) reported a ^29^Si NMR chemical shift of *δ* +29.0, which was interpreted as consistent with delocalization and aromatic character.^30^ Both studies experimentally supported the presence of electron density at silicon *via* reaction with methyl iodide, which led to a mixture of the monomeric 9,9-dimethylsilafluorene, dimeric 9-methylsilafluorene (1), and a siloxane.

We became interested in an alternative synthetic approach to different silafluorene redox states based on electrochemical Si–Si bond activation. An advantage of this approach is the access to not only reduced forms of the silafluorene but also cationic silylium ions *via* oxidation.^31^ Silylium cations play a vital role in various synthetic transformations as reactive intermediates and potent catalysts.^32–35^ Single-electron oxidation or reduction is hypothesized to give rise first to a radical cation or anion that, after Si–Si bond cleavage, would yield the neutral silafluorene radical and either an anion or cation (Fig. 1B). We were particularly interested in characterizing the electronic structure of the ions. This redox study expands on our previous work demonstrating that Si–Si bonds present an exciting platform for novel *neutral* macromolecules due to combining the σ-conjugation and mechanical flexibility.^36,37^

Herein, we report a direct route to dimeric 9-methylsilafluorene 1 in 62% yield that does not require an initial alkali metal reduction (Fig. 1B), and the first X-ray crystal structure of 1. The electrochemical redox behavior of 1 was investigated in THF *via* cyclic voltammetry and showed irreversible reduction and oxidation events. The structure and electronic properties of the reduced and oxidized species, as well as the chemical reactions that might contribute to irreversible redox reactions, were investigated *via* density functional theory (DFT).

## Results and discussion

**Scheme 1:**
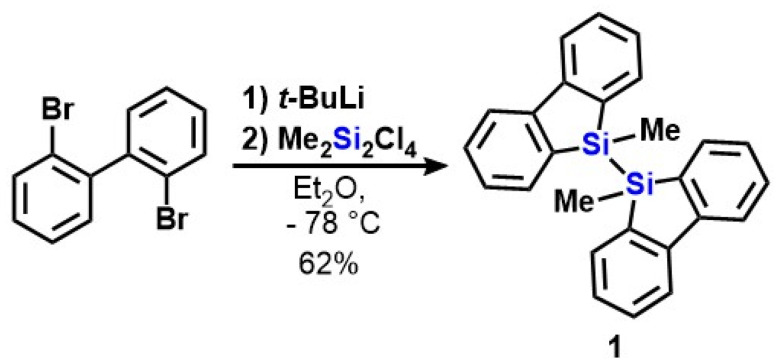
Synthesis of dimeric 9-methylsilafluorene (1).

We first hypothesized that lithium-halogen exchange on 2,2′-dibromo-1,1′-biphenyl followed by salt elimination with 1,1,2,2-tetrachloro-1,2-dimethyldisilane could be used to directly yield the desired dimeric 9-methylsilafluorene (1). Initial attempts with *n*-butyllithium were unsuccessful, presumably due to reaction of the dilithiobiphenyl with bromobutane. Upon switching to *t*-butyllithium, we were then able to obtain compound 1 in 62% yield (Scheme 1). Compound 1 was characterized in solution by a diagnostic ^29^Si NMR chemical shift at *δ* −18.7 in CDCl_3_, in agreement with the previous report,^38^ which also supported its purity as no other silicon resonances were observed.

**Fig. 2:**
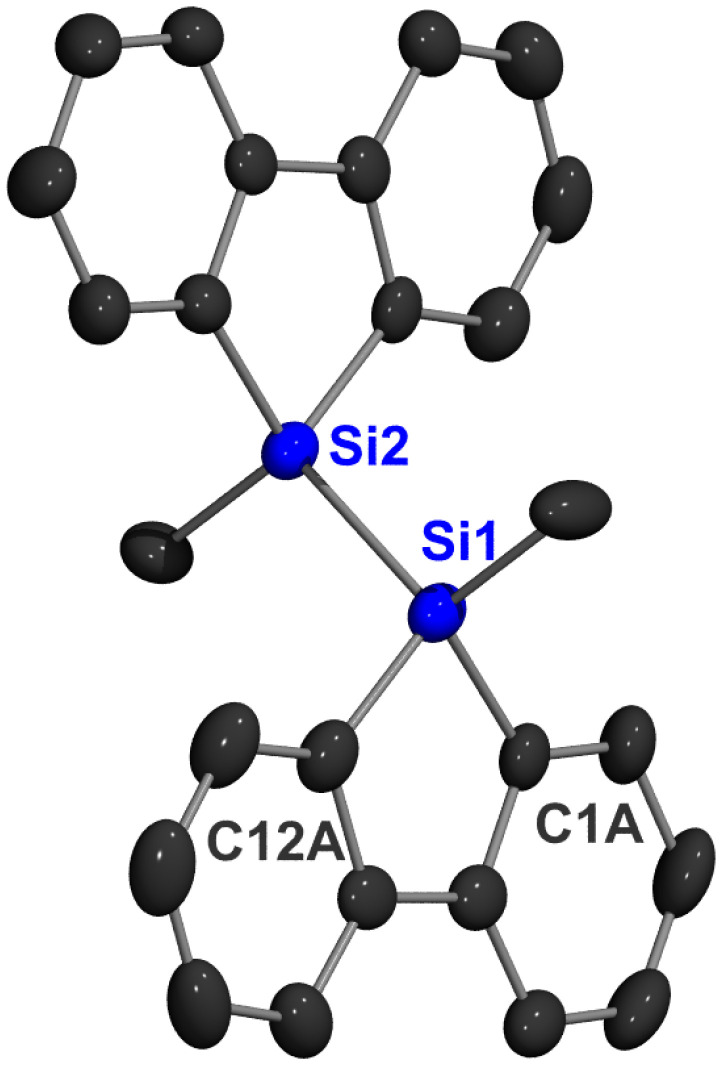
Displacement ellipsoid plot (50% probability level) of one of the two crystallographically independent molecules of 1 at 110.11(10) K. Disorder and H atoms are omitted for clarity. Selected bond lengths [Å]: Si1–Si2 2.3302(17), Si1–C1A 1.877(6), Si1–C12A 1.878(6).

Single crystal X-ray diffraction studies were performed to investigate the structure and bonding of compound 1 (Fig. 2). Colorless rod-shaped crystals of 1 were grown from a concentrated toluene/hexanes (5 : 1) mixture at low temperature. Compound 1 possesses *C*_2h_ symmetry with the Si–Me groups trans to each other. The two crystallographically independent Si1–Si2 and Si3–Si4 bond lengths are 2.3302(17) and 2.3227(15) Å.^30^

**Fig. 3:**
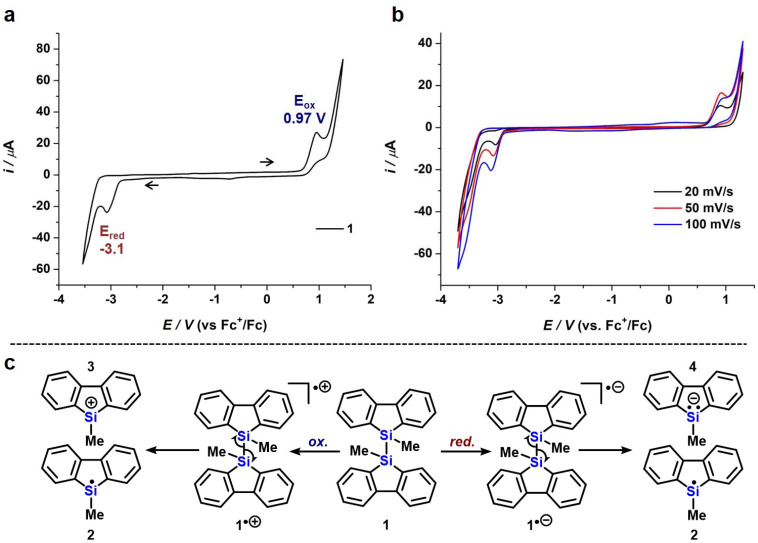
(a) Cyclic voltammogram of a 1.0 mM solution of 1 in THF with 0.1 M TBAPF_6_ at a 100 mV s^−1^ scan rate with a glassy carbon working electrode, graphite counter electrode, and Ag/Ag^+^ as a pseudo-reference electrode. Cyclic voltammograms look identical when scanning in both directions. (b) Scan rate dependence of 1 in the same conditions. Ferrocene was used as an external reference. (c) Hypothesized irreversible decomposition reactions.

Upon isolation of compound 1, we were interested in understanding its electrochemical properties in solution using cyclic voltammetry (CV). We started our CV studies with dichloromethane as the solvent; however, due to the smaller electrochemical solvent window (−1.5 to +1.5 V *vs.* SCE; SCE = saturated calomel electrode)^39,40^ we were unable to observe any redox features. After changing the solvent to THF, which has a slightly larger solvent window (−3.0 to +1.5 V *vs.* SCE),^39,40^ we observe an oxidation wave at 0.97 V *vs.* Fc^+^/Fc and a reduction wave at −3.1 V *vs.* Fc^+^/Fc (Fig. 3a).

Experimental HOMO and LUMO energies were estimated corresponding to eqn (1) and (2), using the *E*_onset_ potentials from cyclic voltammetry.^41^ The HOMO level of 1 was found to be −5.09 eV while the LUMO level was −1.56 eV. From these values, the HOMO–LUMO gap was approximately 3.53 eV, which is within the error of the optical bandgap measured from UV-vis absorption spectroscopy (Table 1). In order to understand the role of rigidity, we investigated the redox behavior of 1,2-dimethyl-1,1,2,2-tetraphenyldisilane (Ph_4_Me_2_Si_2_) as a control, which contains two aromatic substituents on each Si atom but without conformational constraint into a coplanar system. Using the same setup for 1, an irreversible oxidation potential at 0.78 V was observed (Fig. S10†). Unfortunately, we were unable to see a significant reduction feature within the solvent window of THF. This suggests that the LUMO of **Ph**_**4**_**Me**_**2**_**Si**_**2**_ is higher energy than the LUMO of 1. The HOMO of Ph_4_Me_2_Si_2_ was estimated to be −4.98, and the HOMO–LUMO gap was converted to 4.40 eV from the reported optical gap.^42^ We then estimated the LUMO level to be −0.59 eV. The higher oxidation potential of 1 compared to Ph_4_Me_2_Si_2_ suggests a stereoelectronic interaction between Si and the fluorene scaffold that modulates HOMO and LUMO energies (*vide infra*). It is well-known that simple phenyl substituents on silyl radicals and anions are not stabilizing due to the lack of rehybridization between the second-row element Si and the organic system,^43,44^ which could account for the differences between 1 and Ph_4_Me_2_Si_2_.

1 *E*_HOMO_ = −[*E*_ox-onset_ + 4.4]

2 *E*_LUMO_ = −[*E*_red-onset_ + 4.4]

**Table 1:** Tabulated electrochemical data

	*E*_ox_ (V)	*E*_ox-onset_ a (V)	*E*_red_ (V)	*E*_red-onset_ a (V)	*E*_HOMO_ (eV)	*E*_LUMO_ (eV)	HOMO–LUMO gap from CV (eV)	optical gap from UV-vis (eV)
1	0.97	0.69	−3.1	−2.84	−5.09	−1.56	3.53	3.74
Ph_4_Me_2_Si_2_	0.78	0.58	—	—	−4.98	−0.58^a^	—	4.40^b^

a See Fig. S8, S9 and S11† for how the onset potential was measured.

b The HOMO–LUMO gap was calculated from the optical gap of Ph_4_Me_2_Si_2_ using the onset of 282 nm,^42^ and then the *E*_LUMO_ was estimated by subtracting the *E*_HOMO_ from the HOMO–LUMO gap.

The redox features in Fig. 3a were irreversible. A scan rate dependence cyclic voltammetry study at slower rates did not improve reversibility, indicating a rapid electron transfer – chemical (EC) reaction^45^ (Fig. 3b). The irreversible redox feature was hypothesized to reflect Si–Si bond scission forming both the 9-methylsilafluorene radical and the corresponding cation or anion (Fig. 3c).

**Scheme 2:**
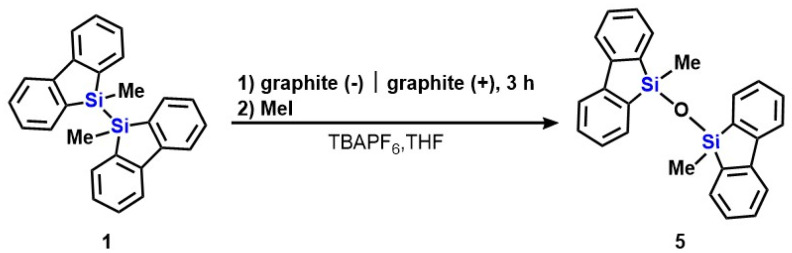
Bulk electroreduction of a 1.0 mM solution of 1 in THF with 0.1 M TBAPF_6_ electrolyte using a graphite cathode and anode and a Ag/Ag^+^ reference electrode. The solution was held at −3.23 V *vs.* Fc^+^/Fc for 3 h and quenched with iodomethane.

We carried out a bulk electrolysis on 1 to isolate and spectroscopically characterize the products formed after reduction (Scheme 2). Since silylium cations have been shown to initiate the cationic ring-opening polymerization of THF,^46–48^ we did not study the oxidation products. The solution of 1 was held at −3.2 V *vs.* Fc^+^/Fc for 3 hours and then quenched with iodomethane. NMR or EPR spectroscopy of the reaction mixture prior to iodomethane quenching was not considered viable due to the short lifetime of silyl radicals at room temperature in the absence of sterically blocking groups.^49^ After extraction from the electrolyte, a ^1^H NMR spectrum confirmed the complete consumption of 1 and the formation of a major product with a singlet at 0.36 ppm (Fig. S13†). Mass spectrometry data were consistent with the known siloxane 5.^30^ A control experiment between 1 and iodomethane in the absence of an electrochemical potential did not produce siloxane 5 and in fact showed no reaction, confirming the role of electrochemical reduction in forming 5 (Fig. S4†). Additionally, chemical reduction of 1 with excess lithium gave a dark green solution consistent with the previous report from Gilman and Gorsich,^28^ and after quenching with H_2_O produced a similar mixture of products obtained from electroreduction including 5 and 9,9-dimetylsilafluorene (Fig. S5†).

While formation of the siloxane 5 supports Si–Si bond cleavage upon reduction, the mechanism by which 5 was formed was not clear. We first considered if the anion 4 or the radical 2 was a likelier precursor to the siloxane 5. West *et al.* reported that siloxane 5 is one of the three products of the reaction between the silafluorene dianion and iodomethane, along with 9,9-dimethylsilafluorene and compound 1, and proposed that 9,9-iodomethylsilafluorene could serve as an intermediate towards siloxane 5 (Fig. S14†). Since methylation of the dianion would give the monoanion 4, it is possible that both reactions proceed through similar intermediates.

However, unlike West, we did not observe the 9,9-dimethylsilafluorene or recover disilane 1. This suggests that the silafluorene radical is a likelier precursor to 5. Possible pathways include (1) hydrogen atom transfer forming the Si–H compound that could undergo autooxidation or (2) iodine atom transfer between the silafluorene radical and iodomethane to give the 9,9-iodomethylsilafluorene, which could hydrolyze to 5 (Fig. S15†). Silyl radicals are known to mediate the reduction of haloalkanes *via* X-atom transfer, supporting the possible formation of siloxane from radical 2.^50–52^

**Fig. 4:**
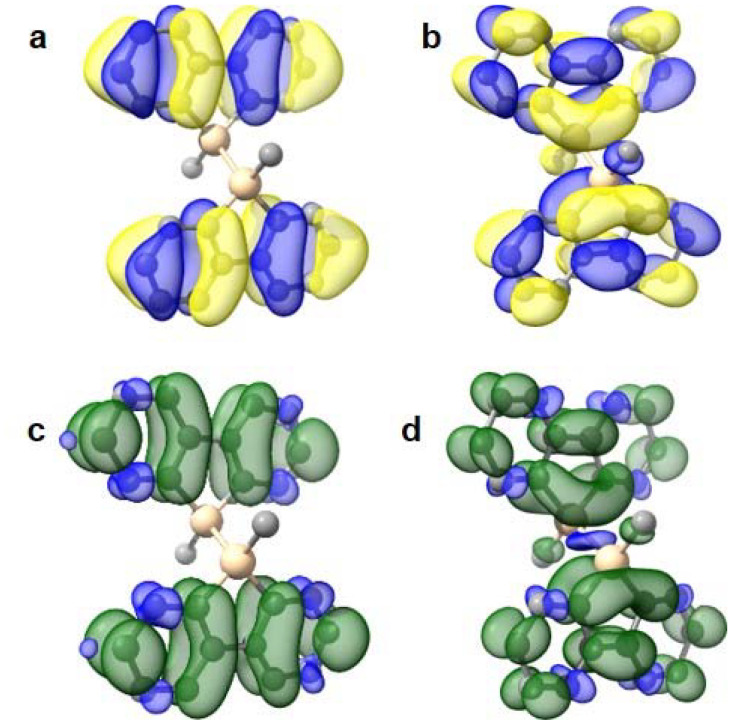
(a) HOMO and (b) LUMO of 1 (isosurface = ±0.003*e* a0-3). Spin density distribution plots of (c) 1˙^+^ and (d) 1˙^−^ (isosurface = ±0.025*e* a0-3). Hydrogen atoms were omitted for clarity.

To assess the impact of rigidification of the silafluorene scaffold on the electronic structure of 1 compared to the unconstrained Ph_4_Me_2_Si_2_, computational modelling was conducted on 1 and its oxidized (1˙^+^) and reduced (1˙^−^) forms. The HOMO and HOMO−1 of 1 comprises a degenerate pair of π-symmetric orbitals, predominantly localized on the annulated biphenyl moieties, with only minor contributions from the C–Si–C bonds of the silole unit of the Si–Si bond (Fig. 4a). In contrast, the LUMO is delocalized over both silafluorene fragments (Fig. 4b). The Si-fluorene interaction in the LUMO of 1 could explain its more facile reduction relative to Ph_4_Me_2_Si_2_. The computed HOMO–LUMO gap of 3.59 eV is in agreement with the experimentally determined value of 3.53 eV. Further insight into the electronic structure were obtained from Hirshfeld atomic population and natural bonding orbital (NBO) analyses, which revealed a partial positive charge of +0.223*e* on each silicon atom and a Wiberg bond index (WBI) of 0.874 for the Si–Si bond (2.339 Å). Consistent with these bonding characteristics, the homolytic bond dissociation energy (*D*_0_) of the Si–Si bond in 1 was calculated to be +75 kcal mol^−1^, aligning with typical values reported for aryl disilane species.^53^

The computed properties and energies of 1 serve as a reference for assessing the effects of redox processes on its electronic structure. Oxidation of 1 to 1˙^+^ leads to a negligible increase of 0.004 Å in the Si–Si bond length (WBI = 0.862); however, the C–C backbone of the silole moieties contracts significantly compared to 1 (Δ*r*(C–C) = −0.063 Å). Despite these structural changes, the calculated homolytic dissociation energy (*D*_0_) for 1˙^+^ suggests that the lability of the Si–Si bond remains largely unaffected, with a value of +72 kcal mol^−1^. Spin density distribution plots for 1˙^+^ reveal that spin density is primarily localized on the annulated phenyl groups of the silafluorene moieties, further suggesting that the local bonding environment of the silicon centers remains relatively unchanged.

In contrast, reduction to 1˙^−^ results in a significant elongation of 0.030 Å in the Si–Si bond, accompanied by a corresponding decrease in the WBI value to 0.782, both indicative of increased lability of the Si–Si bond. Spin density plots for 1˙^−^ show delocalization across the entire silafluorene moiety, with notable α-spin population on both silicon atoms and β-spin accumulation along the Si–Si bond, arising from partial occupation of the Si–Si σ* orbital. NBO analysis confirms the enhanced lability, showing an increase in Si–Si σ* orbital occupation from 0.057*e* in 1 to 0.091*e* in 1˙^−^. Second-order perturbation analysis estimates a destabilization of +4.39 kcal mol^−1^ for the Si–Si bond in 1˙^−^, while the reduced bond dissociation energy for homolytic cleavage (*D*_0_ = +43 kcal mol^−1^) confirms the increased lability.

## Conclusions

In summary, we report the first direct synthesis, X-ray structure, and redox behavior of dimeric 9-methylsilafluorene. In contrast to simply Ph substituents, the fluorene scaffold engages in an electronic interaction with the central Si atom, resulting in more facile electrochemical reduction. The presence of irreversibility in the electrochemical studies suggests the activation of the Si–Si bond. Bulk electroreduction supported our hypothesis of Si–Si bond dissociation upon reduction or oxidation by the formation of a new silafluorene siloxane (2) *via in situ* generation of a silafluorene radical. DFT calculations also supplement this hypothesis through an increase in Si–Si bond length and a decrease in bond dissociation energy after reduction. These data suggest that incorporation of dimeric 9-methylsilafluorene units into other molecular systems will have a substantial effect on the overall electronic behavior and motivate the design of dissociative polymeric scaffoldings with heavier carbon analogues, which are current synthetic targets in our laboratory.

## Author contributions

K. E. W. and R. S. K. conceived the project. K. E. W. synthesized and characterized all compounds and drafted the manuscript. A. M. conducted the computational modeling and data analysis. G. Q. J. performed the electrochemical experiments. V. S. T. analyzed and provided feedback on the electrochemical data. M. A. S. collected and solved the single crystal X-ray diffraction data. D. J. D. W. acquired financial support for the computational modeling. All authors revised the manuscript.

## Conflicts of interest

There are no conflicts to declare.

## Supplementary Material

DT-054-D5DT00097A-s001

DT-054-D5DT00097A-s002

DT-054-D5DT00097A-s003

## Data Availability

The data supporting this article have been included as part of the ESI.†
