# Supplementary material for: Synthesis and redox behavior of Si–Si dimeric 9-methylsilafluorene
Source: Dalton Trans. 2025 Mar 14;54(15):6167–73. doi: 10.1039/d5dt00097a (PMC12039317; doi:10.1039/d5dt00097a)
Supplement: DT-054-D5DT00097A-s001 [file DT-054-D5DT00097A-s001.pdf]

Supplementary Information (SI) for Dalton Transactions.
This journal is © The Royal Society of Chemistry 2025

Supplemental Information

For

Synthesis and Redox Behavior of Si–Si Dimeric 9-Methylsilafluorene

Kelsie E. Wentz,[a]* Andrew Molino,[b] George Q. Jiang,[a] Maxime A. Siegler,[a] David J. D. Wilson,[c] V. Sara Thoi,[a,d] Rebekka S. Klausen[a]*

[a]Department of Chemistry, Johns Hopkins University, 3400 N. Charles St, Baltimore, MD 21218, United States

[b]Department of Chemistry, Massachusetts Institute of Technology, 77 Massachusetts Avenue, Cambridge, Massachusetts 02139, United States

[c]Department of Biochemistry and Chemistry, La Trobe Institute for Molecular Science, La Trobe University, Melbourne, 3086, Victoria, Australia

[d]Department of Materials Science and Engineering, Johns Hopkins University, 3400 N. Charles St. Baltimore, MD 21218 United States

*Correspondence to: kwentz2@jhu.edu or klausen@jhu.edu

**Table of Contents**	**Pg**
General Procedures	S2
Experimental Procedures	S2
NMR Spectra	S4
Cyclic Voltammetry Data	S9
Possible Reaction Pathways with Iodomethane	S17
UV-vis Data	S19
Single Crystal X-Ray Crystallography Data	S20
Theoretical Calculations	S23
References	S24

General Procedures:

Materials: 2,2'-dibromo-1,1'biphenyl was prepared according to the previous literature.[1] 1,1,2,2-tetrachloro-1,2-dimethyldisilane was purchased from TCI. Tert-butyllithium and lithium granules were purchased from Sigma Aldrich. 1,2-dimethyl-1,1,2,2-tetraphenyldisilane was purchased from Ambeed. Tetrabutylammonium hexafluorophosphate was purchased from TCI and recrystallized from methanol prior to use for electrochemical experiments. All other chemicals were used as received. Compound **1** was synthesized under an argon atmosphere using standard Schlenk techniques. Glassware for air- and moisture-sensitive experiments was oven-dried overnight at 175 °C. Diethyl ether and tetrahydrofuran (THF) were dried on a J. C. Meyer Solvent Dispensing System using stainless steel columns packed with neutral alumina.

Instrumentation: NMR spectra were recorded at room temperature on a Bruker Avance 400 MHz spectrometer. The proton and carbon chemical shifts are reported in ppm and referenced using the residual proton and carbon signals of the deuterated solvent (^{1}H: $CDCl_3$ – δ = 7.26; ^{13}C: $CDCl_3$ – δ = 77.2), while the silicon chemical shifts were calibrated by tris(trimethylsilyl)methane in $CDCl_3$. High-resolution mass spectrometry was performed in the Department of Chemistry at Johns Hopkins University using a VG Instruments VG70S/E magnetic sector mass spectrometer with electron ionization (EI) (70 eV). UV-vis spectroscopy data was collected on a Hewlett-Packard Agilent 8453 photodiode-array spectrophotometer of a solution of compound **1** in THF in a 1 cm quartz cuvette. Cyclic voltammetry (CV) experiments measurements were conducted on a Ivium-n-STAT Multichannel Electrochemical Analyzer that was connected to a three-electrode cell using a glassy carbon working electrode, Ag/Ag^+ reference electrode, and graphite counter electrode.

Safety Note: **CAUTION!** t-Butyllithium is highly **pyrophoric** and requires extreme care and accuracy when handling.

Synthesis of Compound 1: In a 100 mL Schlenk flask, 2,2'-dibromo-1,1'-biphenyl (500 mg, 1.60 mmol) was dissolved in dried Et_2O (40 mL) and cooled to -78 °C. t-butyllithium (3.78 mL, 6.41 mmol, 1.7 M in pentane) was then added dropwise, and the resulting solution was stirred at low temperature for 60 min. 1,1,2,2-tetrachloro-1,2-dimethyldisilane (0.244 g, 1.07 mmol) was then added and the reaction was allowed to gradually warm to room temperature and stir for 20 hrs. The reaction was quenched with water (20 mL), and the aqueous layer was extracted with Et_2O (3 x 20 mL). The combined organic layers were dried over Na_2SO_4 and the solvent removed on rotovap to leave a crude colorless oily residue. The residue was redissolved in minimal toluene/hexanes (5:1) for recrystallization at low temperature (259 mg, 62% yield). ^{1}H NMR (400 MHz, $CDCl_3$, 298K) δ 7.88 (d, J = 7.8 Hz, 4H, Ar*H*), 7.61 (d, J = 7.1 Hz, 4H, Ar*H*), 7.46 (t, J = 7.6 Hz, 4H, Ar*H*), 7.29 (t, J = 7.2 Hz, 4H, Ar*H*), 0.24 ppm (s, 6H, Si-C*H*$_3$). ^{13}C NMR (101 MHz, $CDCl_3$) δ 148.4 (Ar*C*), 138.4 (Ar*C*), 133.5 (Ar*C*), 130.0 (Ar*C*), 127.3 (Ar*C*), 121.3 (Ar*C*), -6.13 ppm (Si-*C*H$_3$). ^{29}Si NMR (79 MHz, $CDCl_3$) δ -18.7 ppm. High Resolution MS (EI) Calcd for $C_{26}H_{22}Si_2$: 390.12601, found 390.12565.

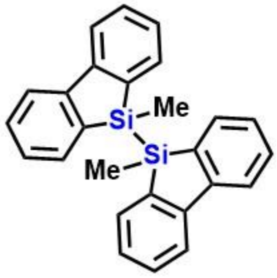

Reduction of 1 with Li: In a 20 mL vial, **1** (67.4 mg, 0.173 mmol) and 18-crown-6 (91.1 mg, 0.345 mmol) were dissolved in 1 mL of THF. Lithium granules (15.0 mg, 2.16 mmol) were then added and stirred vigorously at room temperature. After 5 minutes, the solution turned dark red-orange, and then dark green after 1 hr. The solution was stirred at room temperature for 20 hrs. The THF was removed under reduced pressure, and the dark solids were quenched by washing with hexanes (3 x 5 mL), and dried under vacuum to leave a crude yellow solid (71.0 mg of mixture of products, 45% yield by mass recovery).

NMR Spectra (^{1}H, ^{13}C, and ^{29}Si):

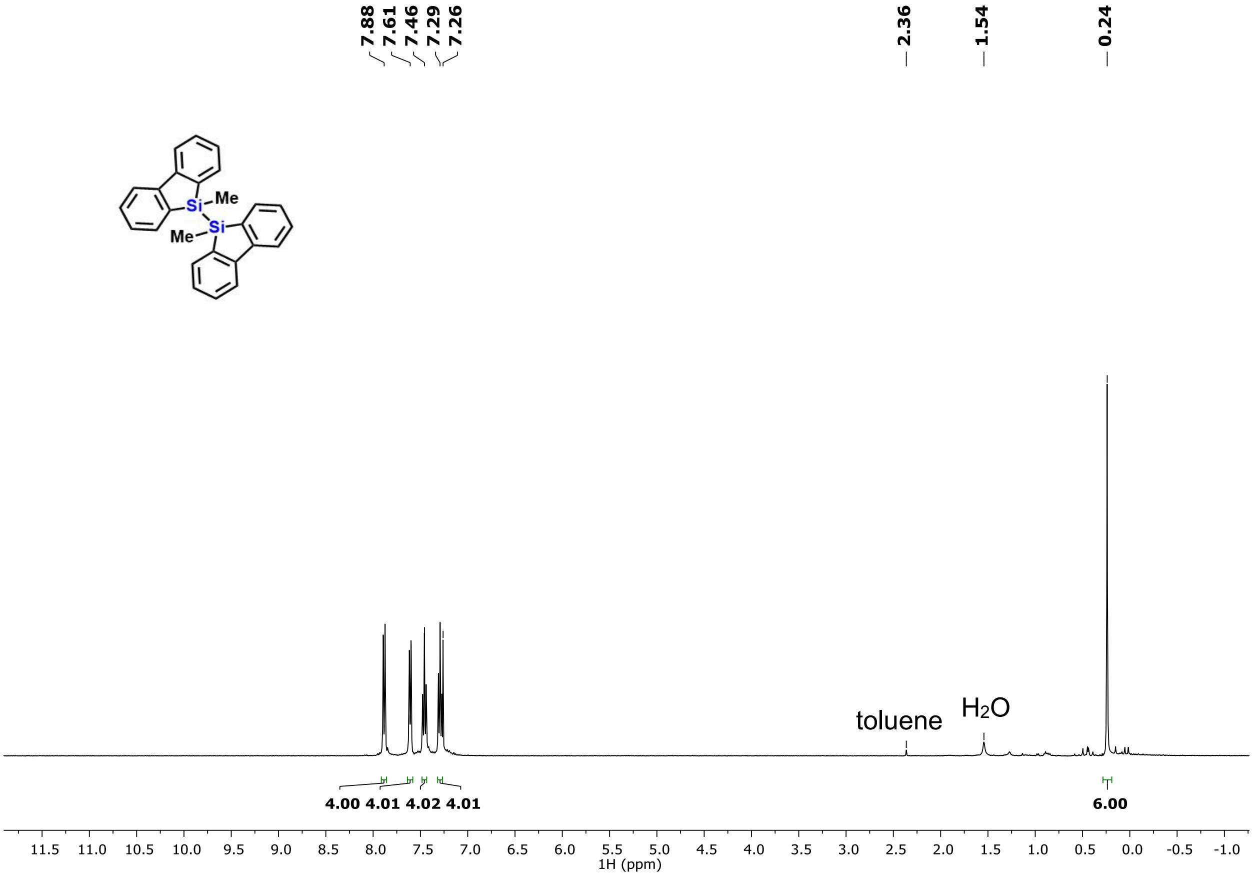

Figure S1. ^{1}H NMR (400 MHz, $CDCl_3$, 298K) spectrum of **1**.

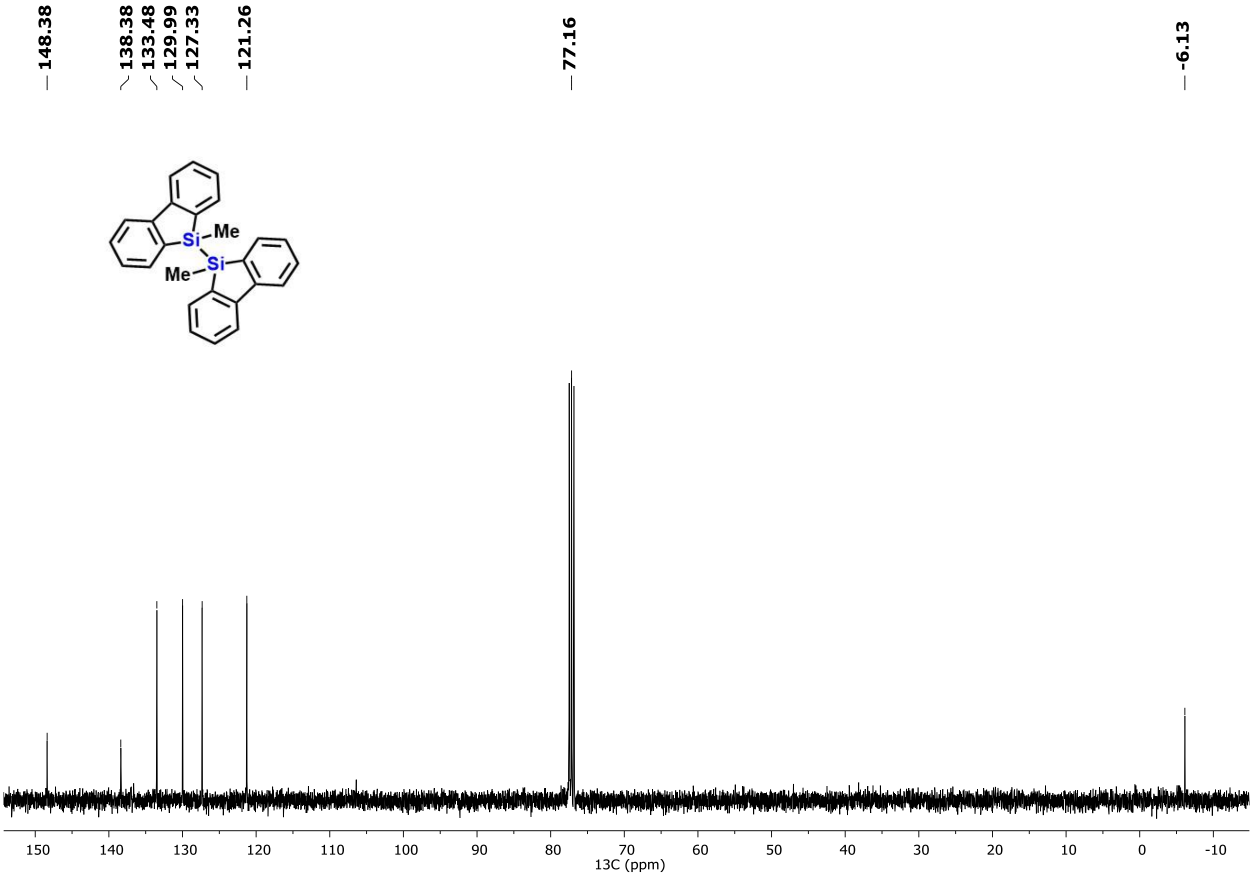

Figure S2. ^{13}C NMR (101 MHz, $CDCl_3$, 298K) spectrum of **1**.

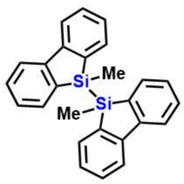

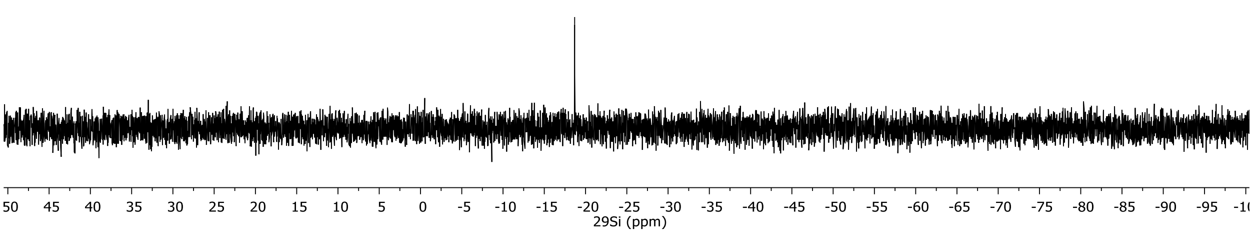

Figure S3. ^{29}Si NMR (79 MHz, $CDCl_3$, 298K) spectrum of **1**.

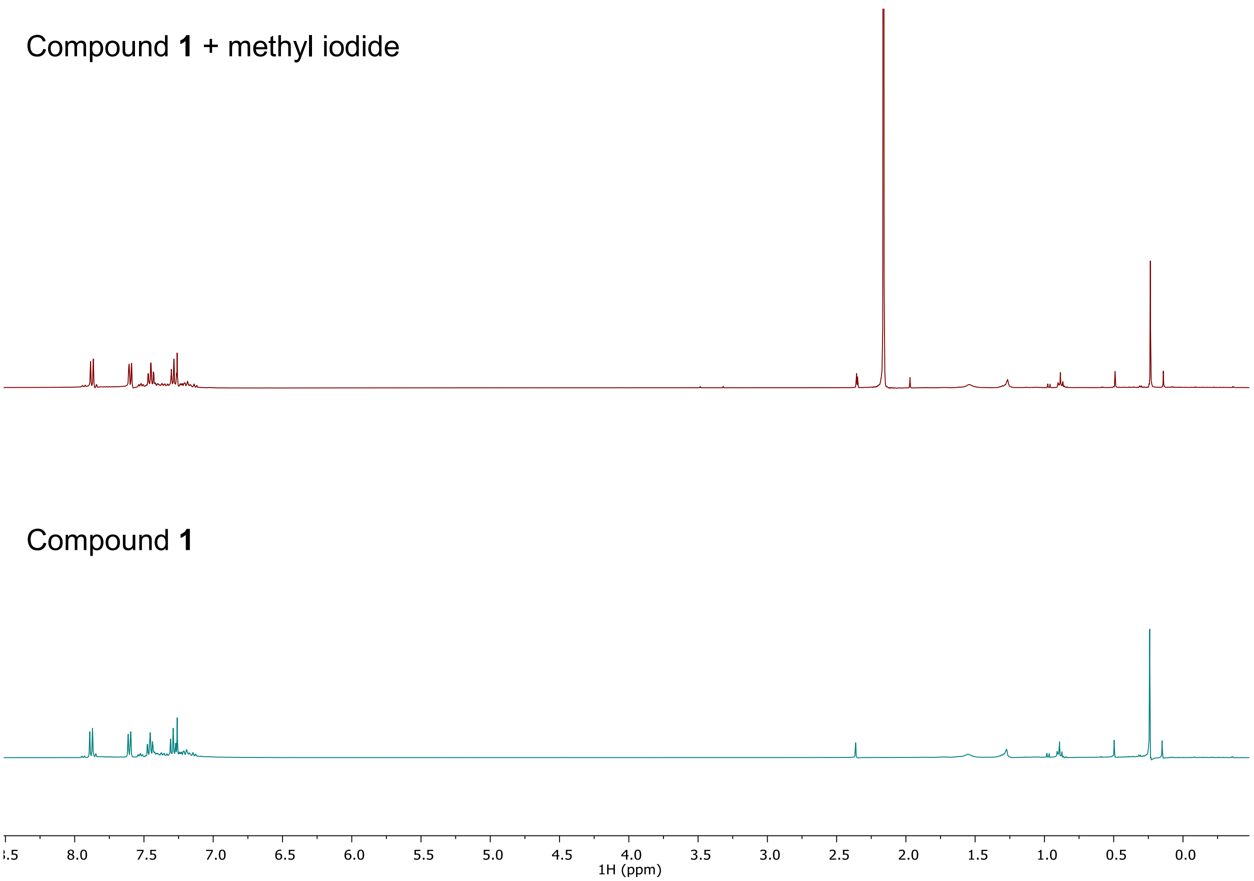

Figure S4. ^{1}H NMR (400 MHz, $CDCl_3$, 298K) spectra of **1** + methyl iodide.

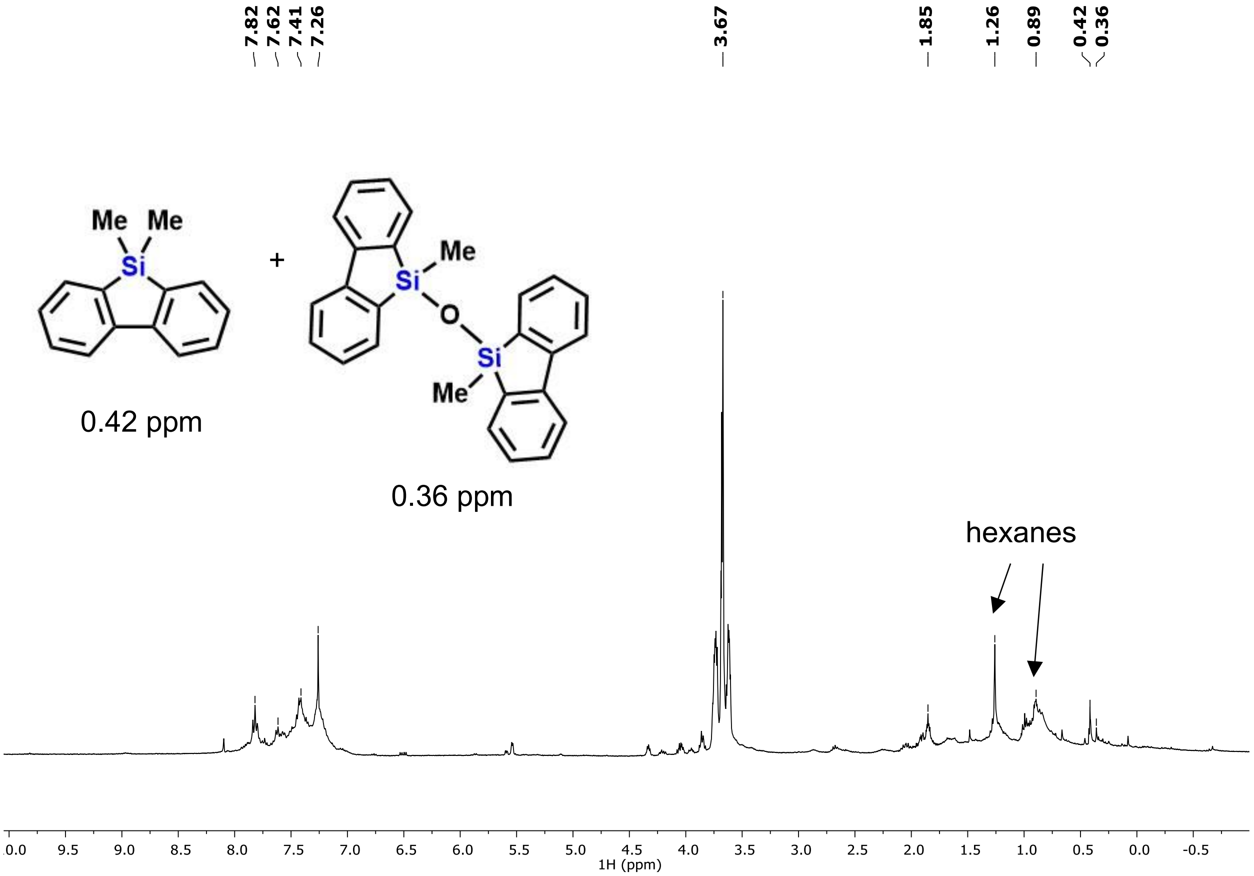

Figure S5. ^{1}H NMR (400 MHz, $CDCl_3$, 298K) spectrum of lithium reduction of **1** after aqueous quench.

Cyclic Voltammetry Data:

Cyclic voltammetry experiments were conducted using tetrabutylammonium hexafluorophosphate (0.1 M in THF) as the supporting electrolyte and 1.0 mM of sample. All electrochemical data was referenced against the ferrocenium/ferrocene oxidation couple in THF ($E_{1/2}$ = 0.54 V). A three-electrode cell in a 3-neck round bottom flask was used with a 3 mm diameter glassy carbon working electrode, Ag/Ag^{+} reference electrode, and graphite counter electrode. The working electrode was polished in sequence with alumina suspensions of 1, 0.3 and 0.05 μm particle sizes on a polishing pad (20 x figure-eight polishing motions per slurry). The system was completely degassed with argon for 15 minutes to remove any oxygen that was introduced during experimental setup. Before CV data of each sample was collected, a blank scan was run to ensure electrodes were properly polished and all the oxygen was removed.

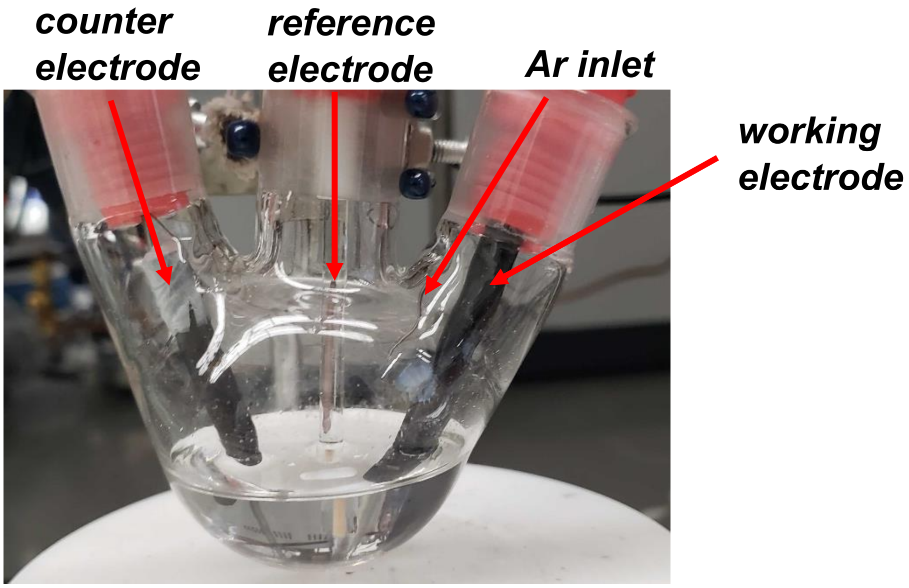

Figure S6. Image depicting setup used for cyclic voltammetry experiments.

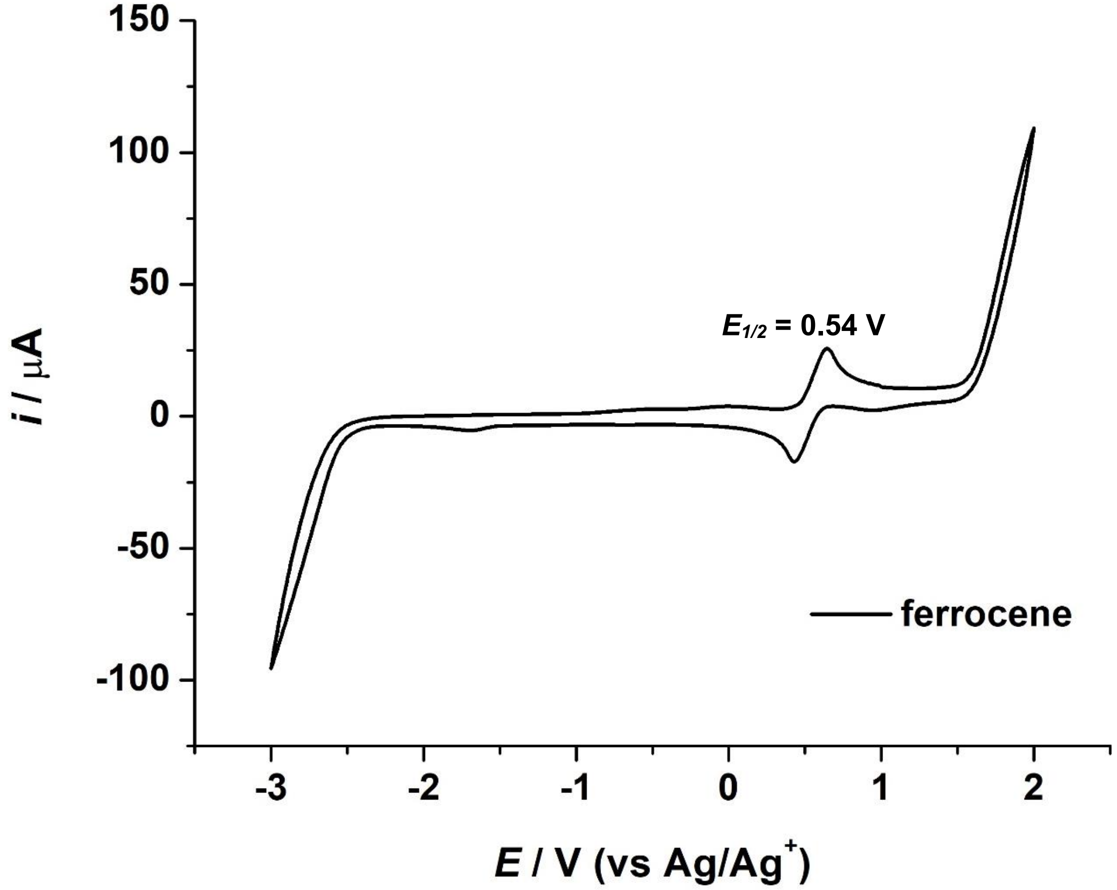

Figure S7. Cyclic voltammogram of 1.0 mM solution of ferrocene in THF with 0.1 M $TBAPF_6$ at 100 mV s^{-1} scan rate vs Ag/Ag^+ reference electrode, with glassy carbon working electrode and graphite counter electrode.

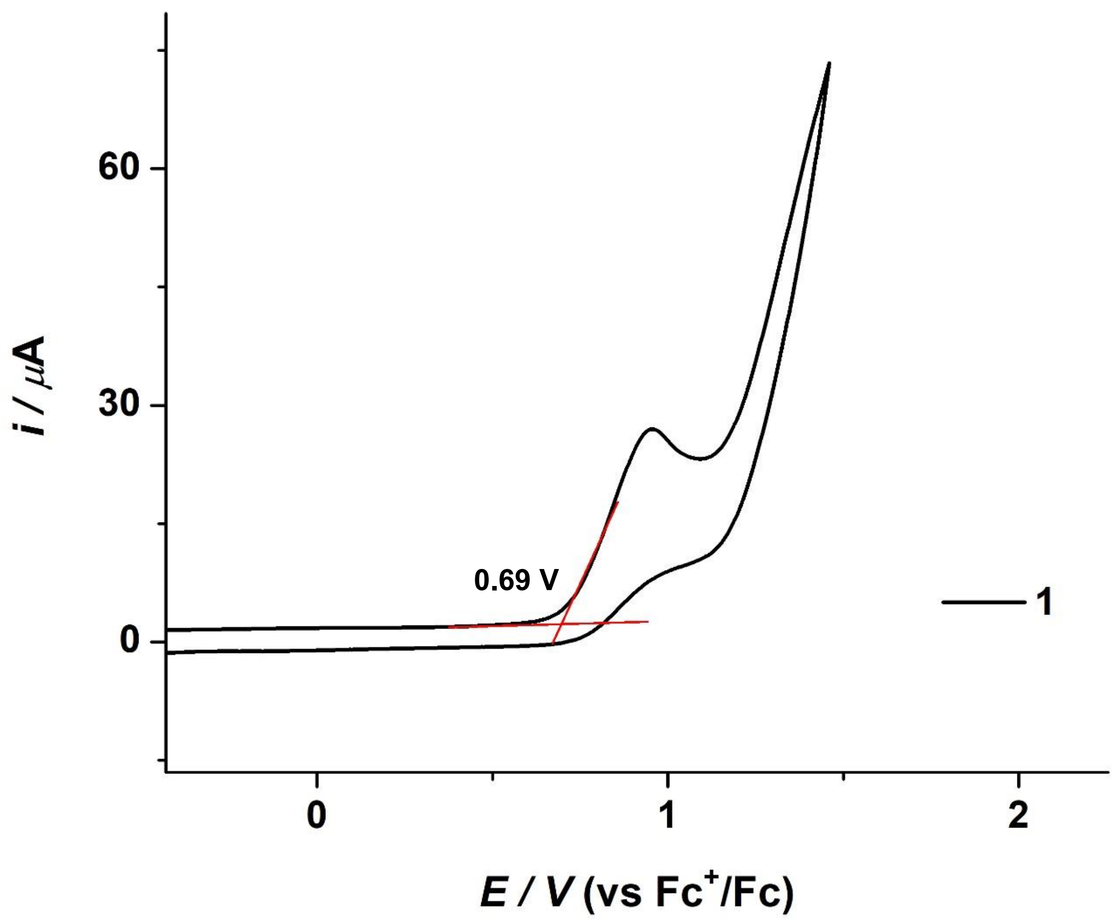

Figure S8. Oxidation onset of 1.0 mM solution of **1** in THF with 0.1 M $TBAPF_6$ at 100 mV s^{-1} scan rate vs Ag/Ag^+ reference electrode, with glassy carbon working electrode and graphite counter electrode.

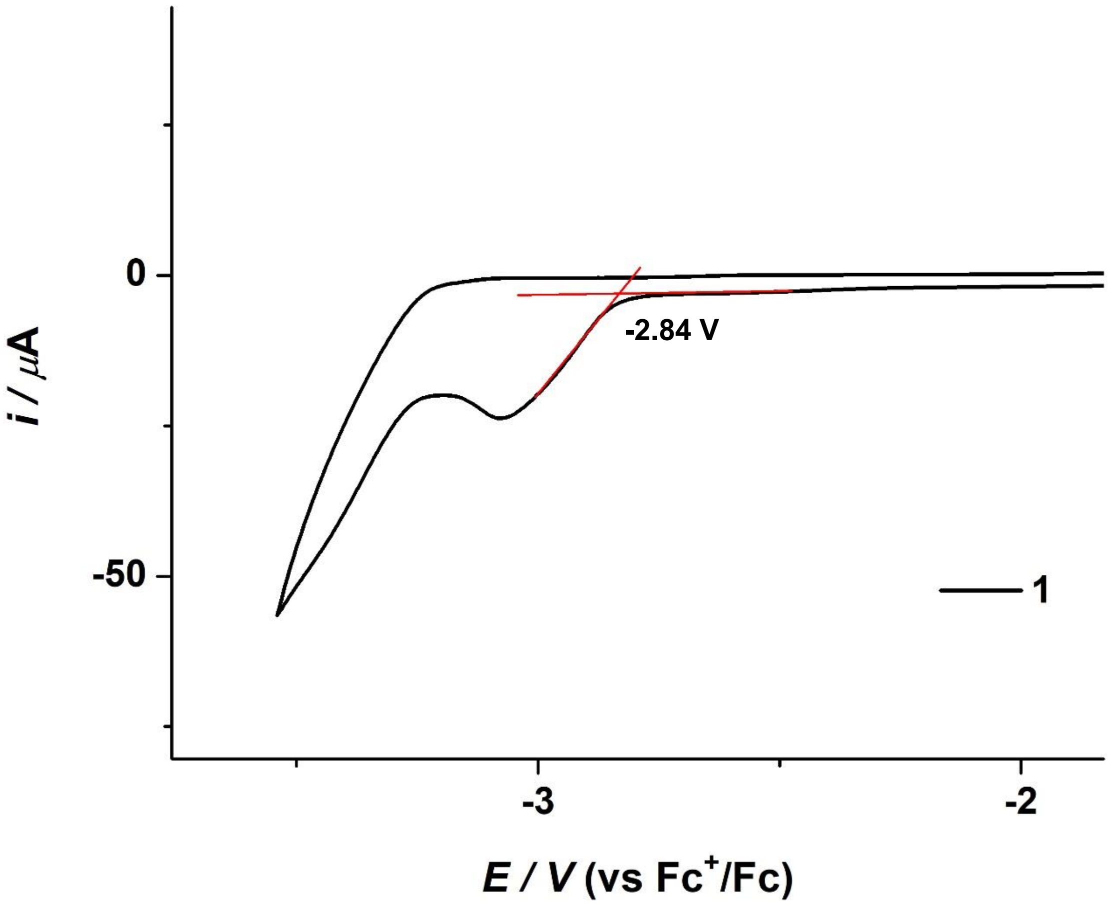

Figure S9. Reduction onset of 1.0 mM solution of **1** in THF with 0.1 M $TBAPF_6$ at 100 mV s^{-1} scan rate vs Ag/Ag^+ reference electrode, with glassy carbon working electrode and graphite counter electrode.

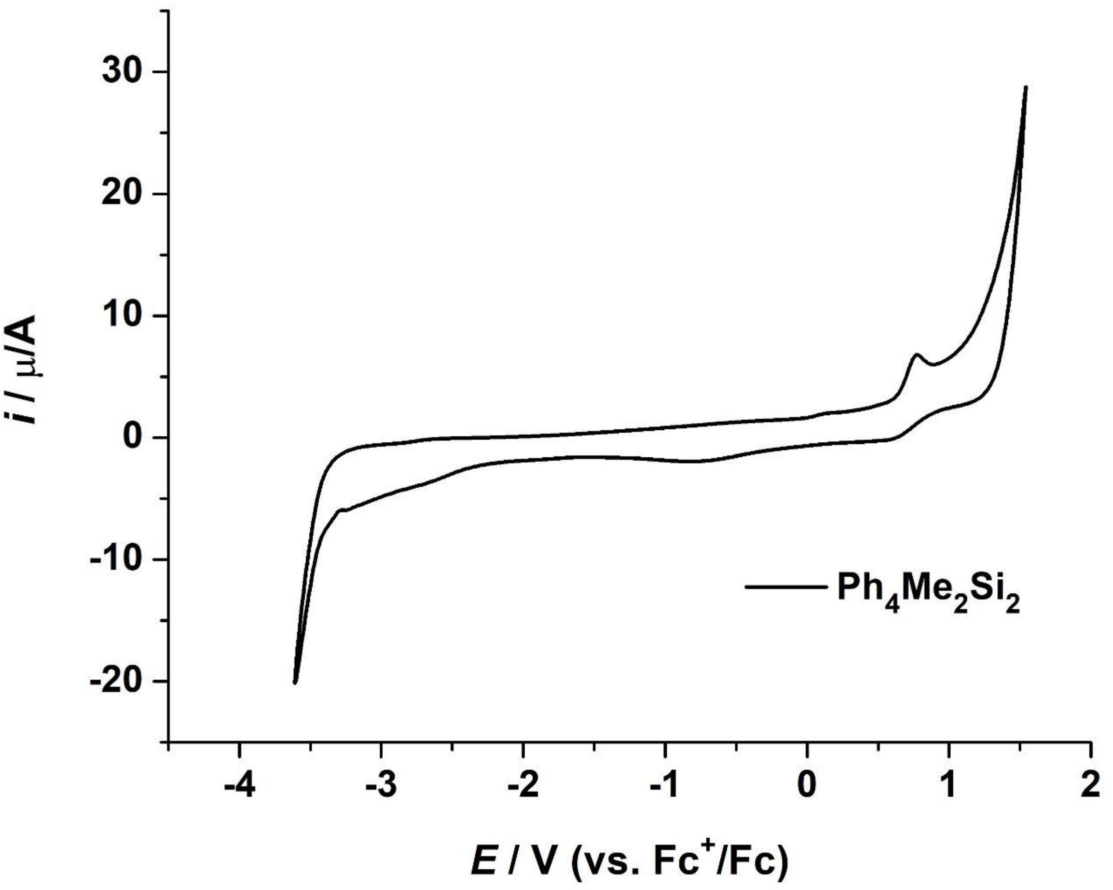

Figure S10. Cyclic voltammogram of 5 mM solution of $\mathbf{Ph_4Me_2Si_2}$ in THF with 0.1 M $TBAPF_6$ at 100 mV s^{-1} scan rate vs Ag/Ag^+ reference electrode, with glassy carbon working electrode and graphite counter electrode.

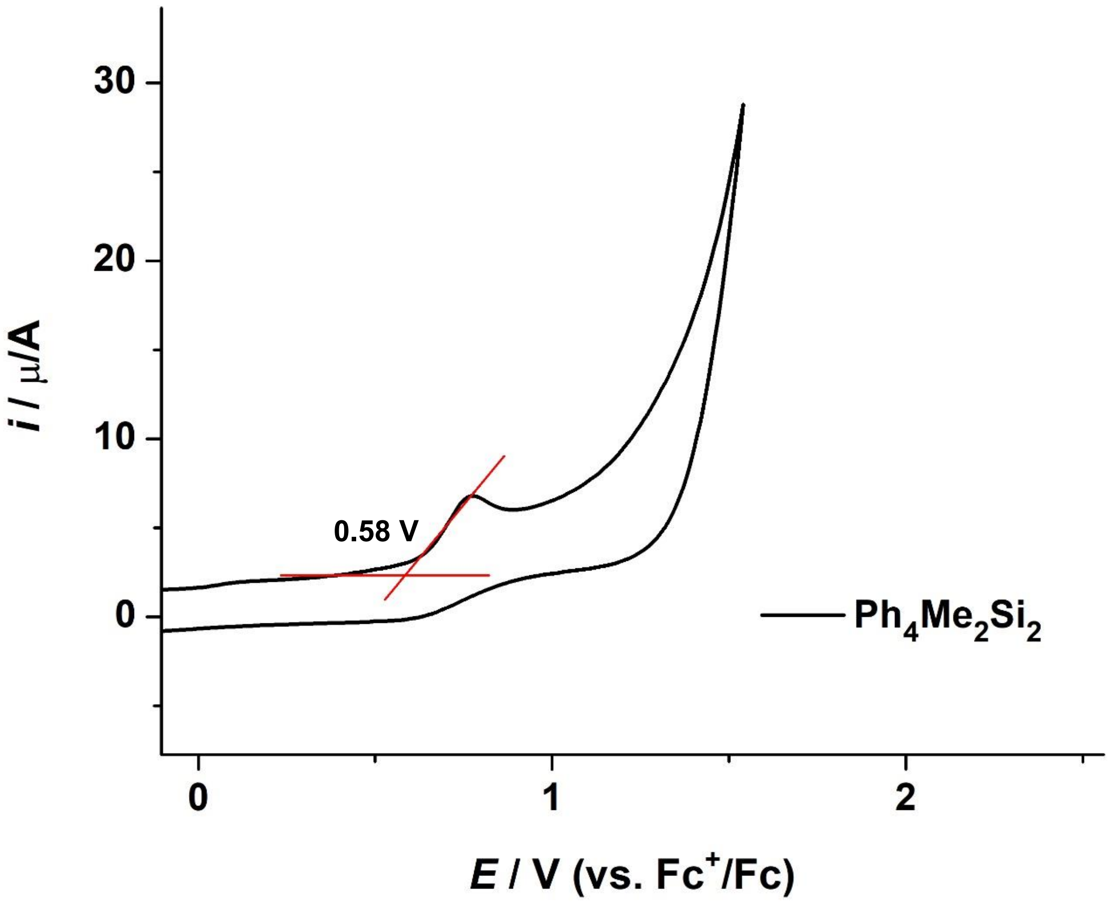

Figure S11. Oxidation onset of 5 mM solution of **$Ph_4Me_2Si_2$** in THF with 0.1 M $TBAPF_6$ at 100 mV s^{-1} scan rate vs Ag/Ag^+ reference electrode, with glassy carbon working electrode and graphite counter electrode.

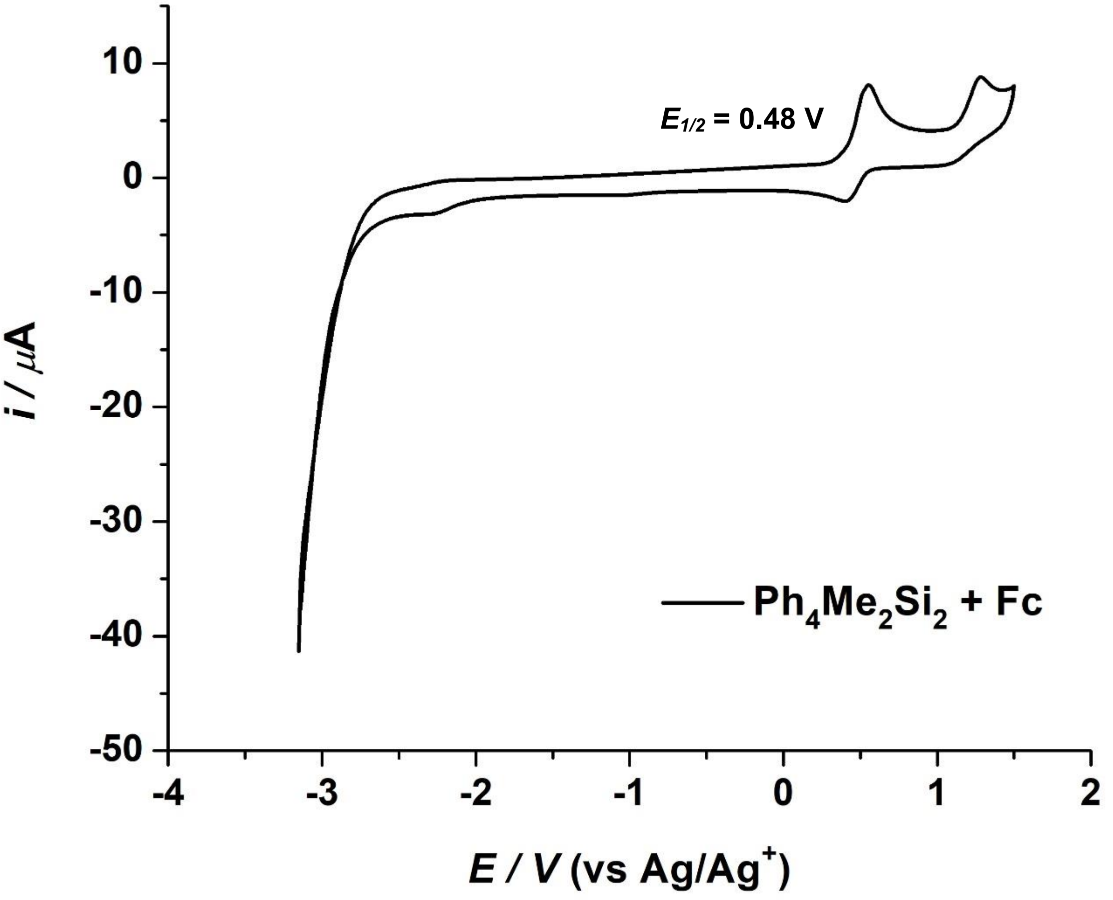

Figure S12. Cyclic voltammogram of 5 mM solution of **$Ph_4Me_2Si_2$** in THF with 0.1 M $TBAPF_6$ at 100 mV s^{-1} scan rate vs Ag/Ag^+ reference electrode, with glassy carbon working electrode and graphite counter electrode.

Bulk Electroreduction of 1:

The bulk electroreduction of **1** was carried out in THF (1 mM) with tetrabutylammonium hexafluorophosphate as the electrolyte (0.1 M), graphite cathode and anode, and Ag/Ag$^+$ reference electrode. The solution was held at a potential of -3.23 V vs Ag/Ag$^+$ for 3 h. After reduction, the solution was quenched with methyl iodide (0.2 mL). The solvent was removed under reduced pressure, and the product was extracted from the electrolyte with hexanes. An NMR spectrum was obtained and used to analyze the products formed from reduction. The product was further characterized via high resolution mass spectrometry which supported the formation of the bis-silafluorene siloxane (**2**). High Resolution MS (EI) Calcd for $C_{26}H_{22}OSi_2$: 406.12092, found 406.11966.

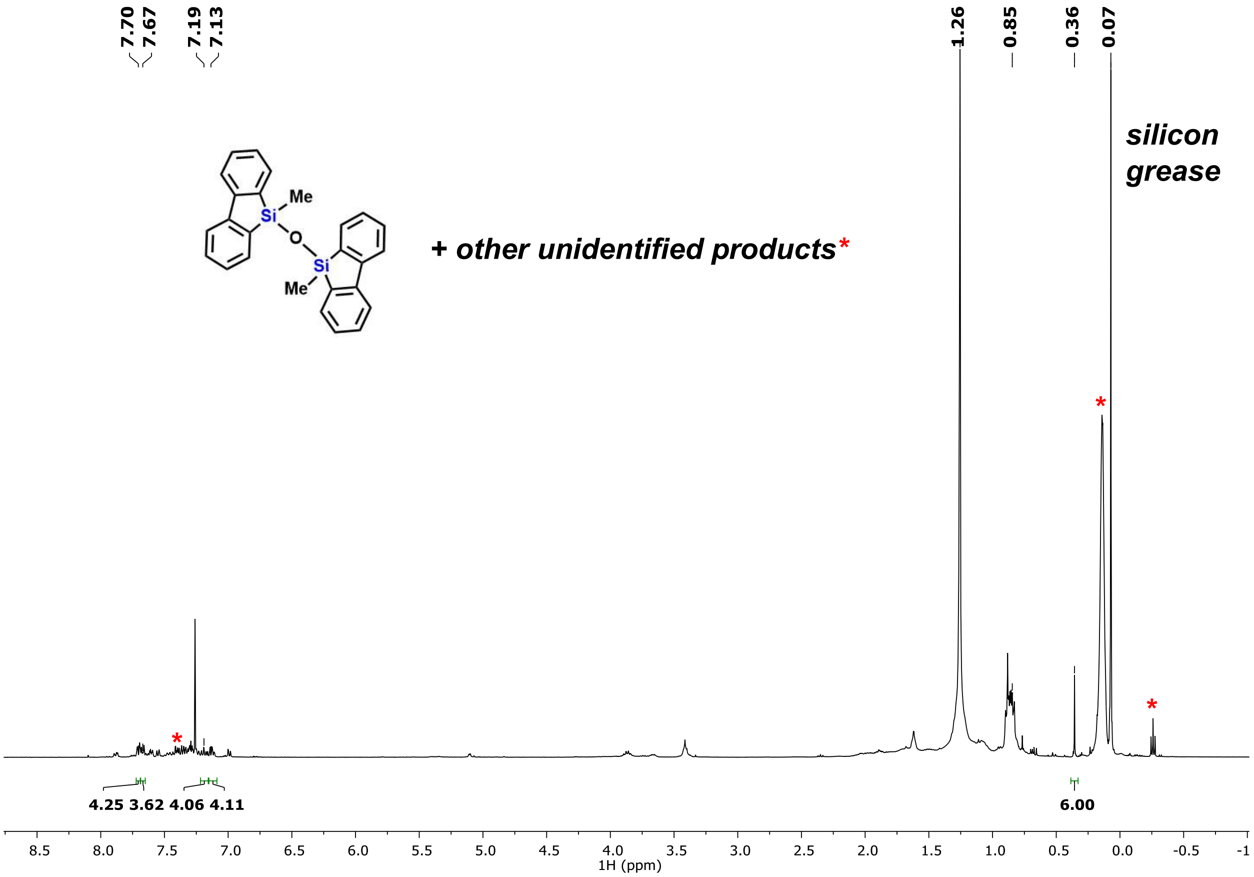

Figure S13. ^{1}H NMR (400 MHz, $CDCl_3$, 298K) spectrum of the extracted solution from the bulk electroreduction of **1**.

Possible Reaction Pathways with Iodomethane:

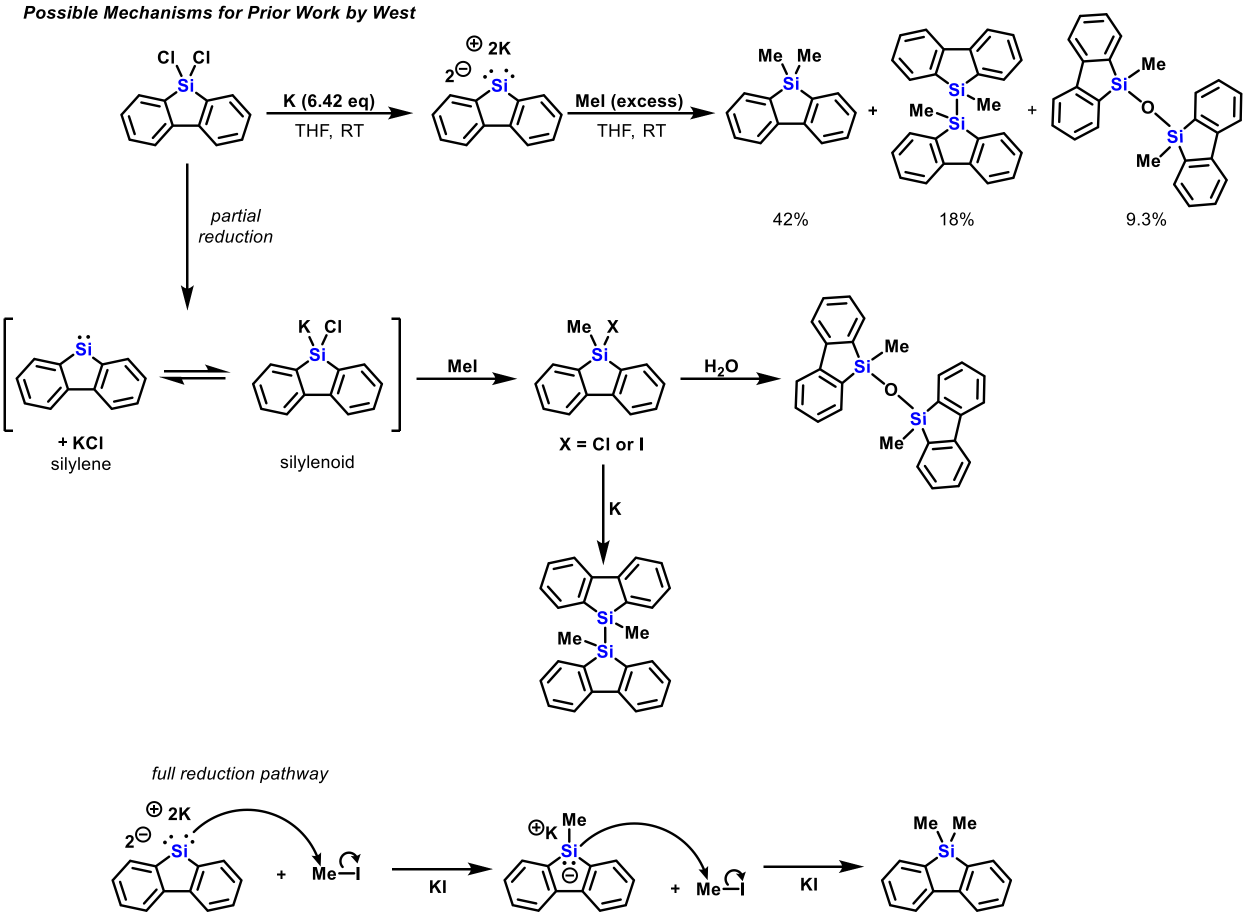

Figure S14. Possible reaction pathway for the partially and double reduced silafluorene with iodomethane to produce the mixture of 9,9-dimethylsilafluorene, dimeric 9-methylsilafluorene, and siloxane.

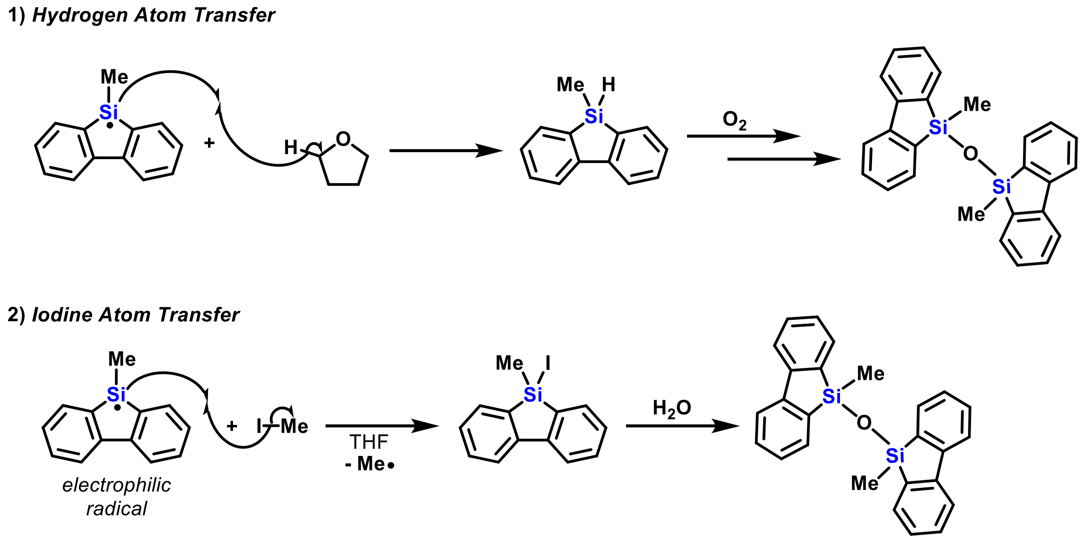

Figure S15. Possible reaction pathways to produce siloxane from silafluorene radical.

UV-vis Data:

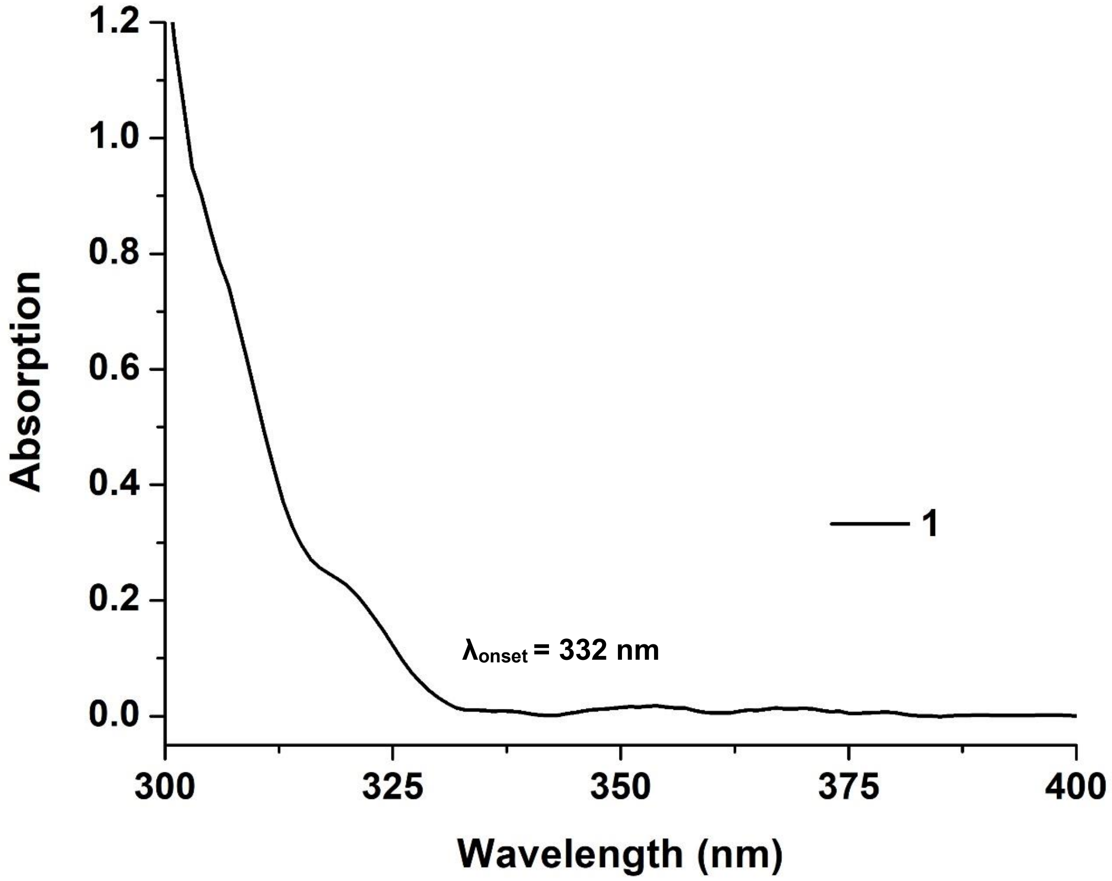

Figure S16. UV-vis spectrum of **1** in THF.

Single Crystal X-Ray Crystallography Data:

All reflection intensities were measured at 110.00(10) K using a Rigaku XtaLAB Synergy R (equipped with a rotating-anode X-ray source and HyPix-6000HE detector) with Cu *K*α radiation (λ = 1.54178 Å) under the program CrysAlisPro (Version CrysAlisPro 1.171.42.49, Rigaku OD, 2022). The same program was used to refine the cell dimensions and for data reduction. The structure was solved with the program SHELXT-2018/2 (Sheldrick, 2018) and was refined on F^2 with SHELXL-2019/3 (Sheldrick, 2018). Analytical numeric absorption correction using a multifaceted crystal model was applied using CrysAlisPro. The temperature of the data collection was controlled using the system Cryostream 1000 from Oxford Cryosystems. The H atoms were placed at calculated positions using the instructions AFIX 43 or AFIX 137 with isotropic displacement parameters having values 1.2 or 1.5 U_{eq} of the attached C atoms.

The structure is disordered. The two crystallographically independent molecules are disordered over two orientations (as both orientations can have very similar space-filling requirements in the crystal), and the major components of the disorder refine to 0.7217(19) and 0.8469(19). The structure is racemically twinned, and the BASF scale factor refines to 0.47(6).

Table S1. Crystallographic Details for Compound 1.

	Compound **1**
Crystal data	
Chemical formula	$C_{26}H_{22}Si_2$
M_r	390.61
Crystal system, space group	Monoclinic, *Cc*
Temperature (K)	110
a, *b*, *c* (Å)	18.4395 (8), 14.6169 (5), 17.3824 (7)
β (°)	112.872 (5)
V (Å^3)	4316.7 (3)
Z	8
Radiation type	Cu *K*α
μ (mm^{-1})	1.54
Crystal size (mm)	0.06 × 0.05 × 0.01
Data collection	
Diffractometer	XtaLAB Synergy R, HyPix
Absorption correction	Analytical<br>*CrysAlis PRO* 1.171.42.95a (Rigaku Oxford Diffraction, 2023) Analytical numeric absorption correction using a multifaceted crystal model based on expressions derived by R.C. Clark & J.S. Reid. (Clark, R. C. & Reid, J. S. (1995). Acta Cryst. A51, 887-897) Empirical absorption correction using spherical harmonics, implemented in SCALE3 ABSPACK scaling algorithm.
T_{min}, T_{max}	0.933, 0.982
No. of measured, independent and observed [$I > 2\sigma(I)$] reflections	33758, 6670, 5529
R_{int}	0.061
$(\sin \Theta/\lambda)_{max}$ (Å^{-1})	0.616
Refinement	
$R[F^2 > 2\sigma(F^2)]$, $wR(F^2)$, S	0.043, 0.108, 1.02
No. of reflections	6670
No. of parameters	852
No. of restraints	1996
H-atom treatment	H-atom parameters constrained

$\Delta\rho_{max}$, $\Delta\rho_{min}$ (e Å^{-3})	0.26, -0.22
Absolute structure	Refined as an inversion twin.
Absolute structure parameter	0.47 (6)

Computer programs: *CrysAlis PRO* 1.171.42.49 (Rigaku OD, 2022), *SHELXT2018*/2 (Sheldrick, 2018), *SHELXL2019*/3 (Sheldrick, 2018), *SHELXTL* v6.10 (Sheldrick, 2008).

Theoretical Calculations:

All calculations were performed using Orca 5.0.4.[2] Geometry optimizations employed the composite r^{2}SCAN-3C method with single-point calculations performed using the B3LYP hybrid exchange-correlation functional, Grimme's D3 dispersion correction with Becke-Johnson damping, and the def2-TZVP basis set, denoted as B3LYP-D3(BJ)/def2-TZVP.[3-5] All optimizations were conducted in solvent via the conductor-like polarizable continuum model (CPCM) with tetrahydrofuran (THF) parameters.[6] The resolution of identity approximation was applied to both Coulomb and Hartree-Fock exchange integrals, with a 590-point integration grid. To ensure optimized geometries represented minima on the potential energy surface, analytical harmonic frequency calculations were performed. NBO 7.0 was employed to compute atomic spin populations, NPA charges, Wiberg bond indices, and second-order perturbation analysis.[7]

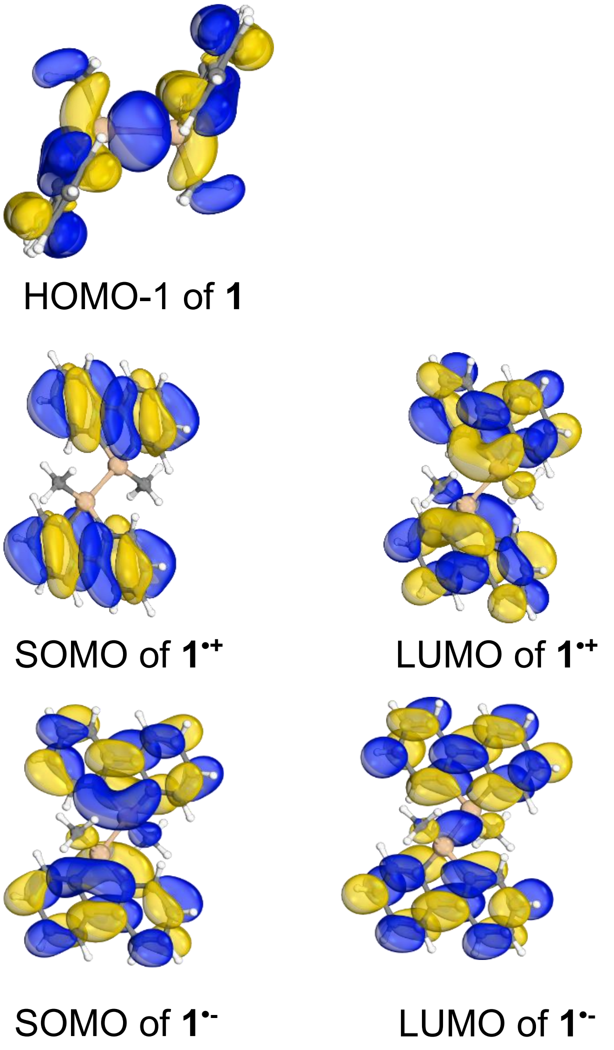

Figure S17. Selected Molecular Orbitals of **1**, $\mathbf{1}^{\bullet+}$, and $\mathbf{1}^{\bullet-}$.

References

1. Chan, K. L.; Watkins, S. E.; Mak, C. S. K.; McKiernan, M. J.; Towns, C. R.; Pascu, S. I.; Holmes, A. B., Poly(9,9-dialkyl-3,6-dibenzosilole)—a high energy gap host for phosphorescent light emitting devices. *Chem. Commun.* **2005,** (46), 5766-5768.

2. Neese, F., Software update: The ORCA program system—Version 5.0. *WIRES Comput. Mol. Sci.* **2022,** *12* (5), e1606.

3. Becke, A. D., Density-functional thermochemistry. III. The role of exact exchange. *J. Chem. Phys.* **1993,** *98* (7), 5648-5652.

4. Grimme, S.; Antony, J.; Ehrlich, S.; Krieg, H., A consistent and accurate ab initio parametrization of density functional dispersion correction (DFT-D) for the 94 elements H-Pu. *J. Chem. Phys.* **2010,** *132* (15).

5. Weigend, F.; Ahlrichs, R., Balanced basis sets of split valence, triple zeta valence and quadruple zeta valence quality for H to Rn: Design and assessment of accuracy. *Phys. Chem, Chem. Phys.* **2005,** *7* (18), 3297-3305.

6. Barone, V.; Cossi, M., Quantum Calculation of Molecular Energies and Energy Gradients in Solution by a Conductor Solvent Model. *J. Phys. Chem. A* **1998,** *102* (11), 1995-2001.

7. Glendening, E. D.; Landis, C. R.; Weinhold, F., NBO 7.0: New vistas in localized and delocalized chemical bonding theory. *J. Comput. Chem.* **2019,** *40* (25), 2234-2241.
